# Geographical distribution of soil transmitted helminths and the effects of community type in South Asia and South East Asia – A systematic review

**DOI:** 10.1371/journal.pntd.0006153

**Published:** 2018-01-18

**Authors:** Zachary A. Silver, Saravanakumar P. Kaliappan, Prasanna Samuel, Srinivasan Venugopal, Gagandeep Kang, Rajiv Sarkar, Sitara S. R. Ajjampur

**Affiliations:** 1 Division of Geographic Medicine and Infectious Diseases, Tufts University School of Medicine, Boston, MA, United States of America; 2 Division of Gastrointestinal Sciences, Christian Medical College, Vellore, India; 3 Department of Biostatistics, Christian Medical College, Vellore, India; University of Kelaniya, SRI LANKA

## Abstract

**Background:**

Soil-transmitted helminth (STH) infections are among the most prevalent neglected tropical diseases (NTD) worldwide. Since the publication of the WHO road map to combat NTD in 2012, there has been a renewed commitment to control STH. In this study, we analysed the geographical distribution and effect of community type on prevalence of hookworm, *Trichuris* and *Ascaris* in south Asia and south east Asia.

**Methodology:**

We conducted a systematic review of open-access literature published in PubMed Central and the Global Atlas of Helminth Infection. A total of 4182 articles were available and after applying selection criteria, 174 studies from the region were retained for analysis.

**Principal findings:**

*Ascaris* was the commonest STH identified with an overall prevalence of 18% (95% CI, 14–23%) followed by *Trichuris* (14%, 9–19%) and hookworm (12%, 9–15%). Hookworm prevalence was highest in Laos, Vietnam and Cambodia. We found a geographical overlap in countries with high prevalence rates for *Trichuris* and *Ascaris* (Malaysia, Philippines, Myanmar, Vietnam and Bangladesh). When the effect of community type was examined, prevalence rates of hookworm was comparable in rural (19%, 14–24%) and tribal communities (14%, 10–19%). Tribal communities, however, showed higher prevalence of *Trichuris* (38%, 18–63%) and *Ascaris* (32%, 23–43%) than rural communities (13%, 9–20% and 14%, 9–20% respectively). Considerable between and within country heterogeneity in the distribution of STH (I^2^ >90%) was also noted. When available data from school aged children (SAC) were analysed, prevalence of *Ascaris* (25% 16–31%) and *Trichuris* (22%, 14–34%) were higher than among the general population while that of hookworm (10%, 7–16%) was comparable.

**Conclusions/Significance:**

Our analysis showed significant variation in prevalence rates between and within countries in the region. Highlighting the importance of community type in prevalence and species mix, we showed that tribal and rural communities had higher hookworm infections than urban communities and for ascariasis and trichuriasis, tribal populations had higher levels of infection than rural populations. We also found a higher prevalence of ascariasis and trichuriasis in SAC compared to the general population but comparable levels of hookworm infections. These key findings need to be taken into account in planning future MDA and other interventions.

## Author summary

Soil transmitted helminth infections (hookworms, *Trichuris* and *Ascaris*) are highly prevalent across south Asia and south east Asia and recently several large initiatives have been launched to control or interrupt transmission. We conducted a systematic review of STH infections to identify the communities in south Asia and south east Asia that will benefit the most from intervention programs. Our analysis showed that *Ascaris* is the most prevalent STH in the region but there was considerable geographic variation in the region. We found that tribal and rural communities in these countries had higher prevalence of STH compared to urban populations. We also found a higher prevalence of *Ascaris* and *Trichuris* in school aged children compared to the general population but comparable levels of hookworm infections. These key findings are important for future planning of intervention strategies for control of STH.

## Introduction

Soil-transmitted helminth (STH) infections are among the most prevalent neglected tropical diseases (NTD) with an estimated 1.45 billion people infected with at least one species worldwide [1, 2]. This number, however, likely remains an underestimate due to the lack of high quality epidemiological data from most geographical regions. Infection by the STH human hookworm (*Ancylostoma duodenale* and *Necator americanus)*, *Trichuris trichiura* (*Trichuris*) and *Ascaris lumbricoides* (*Ascaris*) usually do not result in mortality, but instead lead to chronic infections and extended morbidity. Chronically infected children, for example, have been shown to experience malnutrition, stunting and cognitive deficits [3, 4], while pregnant women develop STH-induced anemia [5, 6]. The “hidden” morbidity of STH infection not only takes a huge toll on the health of an individual, but has also been shown to affect economic development [7, 8]. In 2012, the World Health Organization (WHO) published a comprehensive roadmap to combat NTDs by 2020, and for STHs, had set the goal of achieving 75% mass drug administration (MDA) coverage in all endemic countries [9]. This WHO initiative was further strengthened by the London declaration with a commitment from pharmaceutical companies and other organizations to support global efforts to control or eliminate 10 specific NTDs including STHs. In order to ensure optimal use of these resources and achieve transmission interruption of STHs, it will be critical to target the right communities in these countries for intervention.

A recent review on the global burden of STH infection revealed that nearly 70% of the infections occur in Asia [2]. In the same study, it was found that one quarter (26.4%) of the Asian study population hosted at least one STH species. The high STH burden in Asia is probably due to the moist and tropical climatic conditions, scarcity of safe drinking water, inadequate sanitation, and poor hygiene practices, all of which facilitates worm survival and transmission [10, 11]. What remains unclear, however, is the extent of geographic variability in the prevalence of STH infections between and within countries. Earlier studies have shown that the prevalence of STHs may vary considerably between countries, and also between rural and urban communities [12–14]. Furthermore, since treatment efficacy and reinfection rates are different for each species of STH, in regions with ongoing MDA programs, this would also influence the prevalence and species distribution [15, 16]. In this study, we aimed to assess the geographical distribution of hookworm *Trichuris*, and *Ascaris* in south Asia and south east Asia and to understand the effect of community type, namely urban, rural and tribal on prevalence of STHs.

## Methods

We conducted a systematic review of open-access literature published between January 1, 1990 and June 15, 2015 to assess the association between STH prevalence and community type (rural, urban, or tribal) in south Asia and south east Asia. Countries were included on the basis of membership to the South Asian Association for Regional Cooperation (SAARC) or Association of Southeast Asian Nations (ASEAN). These include the following countries: Bhutan, India, Pakistan, Sri Lanka, Bangladesh, Nepal, Maldives and Afghanistan (SAARC) and Malaysia, Brunei, Cambodia, Laos, Vietnam, Philippines, Myanmar, Indonesia, Singapore and Thailand (ASEAN). This systematic review was performed in accordance with the Preferred Reporting Items for Systematic Reviews and Meta-Analyses (PRISMA) guidelines (S1 Text) [17].

### Search strategy

We searched PubMed Central using the keywords soil transmitted helminth, nematode, *Necator*, *Ancylostoma*, *Ascaris*, *Trichuris*, hookworm, roundworm, and whipworm. The search was further narrowed using the keywords aborigine, aboriginal, tribe, tribal, indigenous, rural, village, town, city, and slum. To supplement our PubMed Central search, we accessed the Global Atlas of Helminth Infection (GAHI—http://www.thiswormyworld.org/) database for all mapped and unmapped studies of STH infection. For India-specific articles, we hand searched India specific journals from the Christian Medical College (CMC), Vellore library.

The articles collected through the searches were evaluated for inclusion in the meta-analysis based on the following criteria: i) only studies containing primary data on STH infection were included (any reviews and meta-analyses that were acquired during our search were separately catalogued), ii) only studies conducted in the eligible countries mentioned above were included and studies conducted in other Asian countries were excluded and iii) as we also planned to determine the prevalence of STH as a function of the type of community environment, we only included studies that clearly defined the study population as rural, tribal, or urban. Based on the description of the study population by the author we categorized the studies as urban /rural /tribal. "Suburban" areas and "towns" were also considered urban. A semi-urban slum was considered "urban", while a rural slum was considered "rural". We excluded studies under "unclear whether rural/urban/tribal" category as we could not find the description of the study population in the article. If a given reference had data from more than one community type, the data was collected and analysed as separate datasets. Studies measuring STH infection in hospital inpatients or outpatients, immunocompromised individuals, or individuals with underlying disease condition(s) were excluded from this analysis.

### Data extraction

For data extraction, two reviewers used a standardized excel data extraction form based on the PRISMA guidelines to determine inclusion eligibility for the articles. For each article, the researchers independently attempted to extract the following data: reference name, country, study year (publication year if study year was not available), location, study population, community type, age group, school aged children (SAC) or not, study design, number of stool samples collected per person, reported prevalence (*Ascaris*, *Trichuris* and hookworm), and sample size. A single reviewer was responsible for comparing the two independent extractions and compiling a list with any discrepancies. These discrepancies were then presented to and resolved by a third reviewer. Data from a particular reference was added to the final STH infection database once both the independent reviewers (three in cases of discrepancy) were in agreement.

### Data analysis

We calculated the prevalence by dividing the number of STH positive individuals by the total number of participants. We then applied a logit transformation to obtain normality and weighted by inverse variance of logit-transformed prevalence. The I^2^ statistic was used to assess heterogeneity between studies [18]. We subsequently performed random-effects model to account for heterogeneity in prevalence estimates. Because infection is highly correlated with community type and age, we stratified the analyses both according to community type (urban, rural and tribal) and whether or not the study included SAC. Data analysis was carried out using R statistical software v. 2.8.1 [19].

### Results

**Fig 1:**
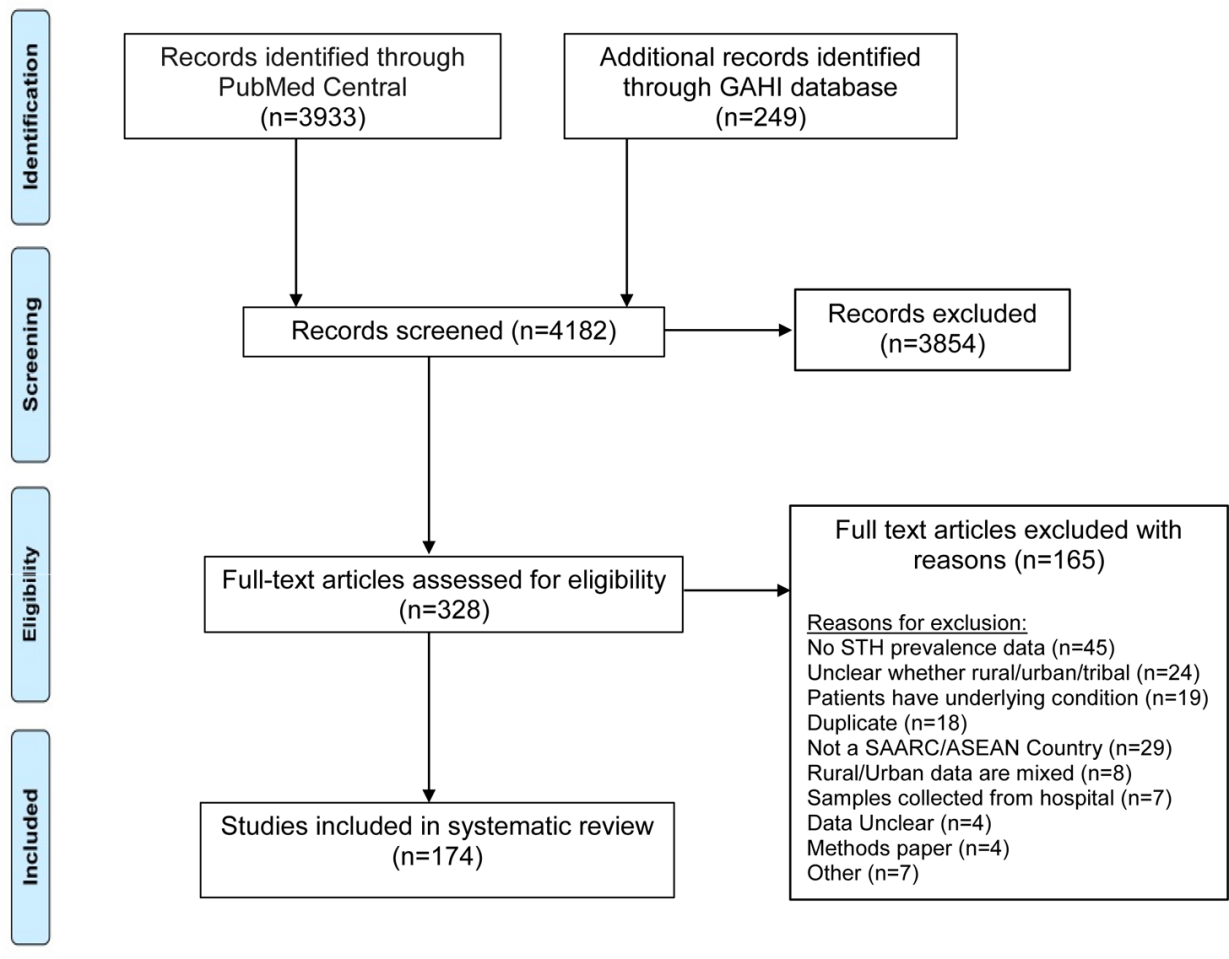
PRISMA flow diagram.

A total of 4182 articles were available based on the search strategy given above and when selection criteria were applied, 3854 records were excluded. Of the remaining 328 full text articles assessed for eligibility, 165 articles were further excluded and 174 articles from 14 countries met the inclusion criteria (Fig 1). Among the 14 countries included in this study, India (46 publications), Malaysia (21 publications) and Thailand (19 publications) had the most contributions. On the other hand, there were no studies from Bhutan, Maldives and Singapore that met the inclusion criteria. The only study from Afghanistan that met the inclusion criteria was carried out on 239 urban SAC with a 57% prevalence of *Ascaris* and 13% prevalence of *Trichuris* and no hookworm; this was excluded from the country specific analysis. A list of all 174 references included in the analysis is provided in S1 Table.

**Fig 2:**
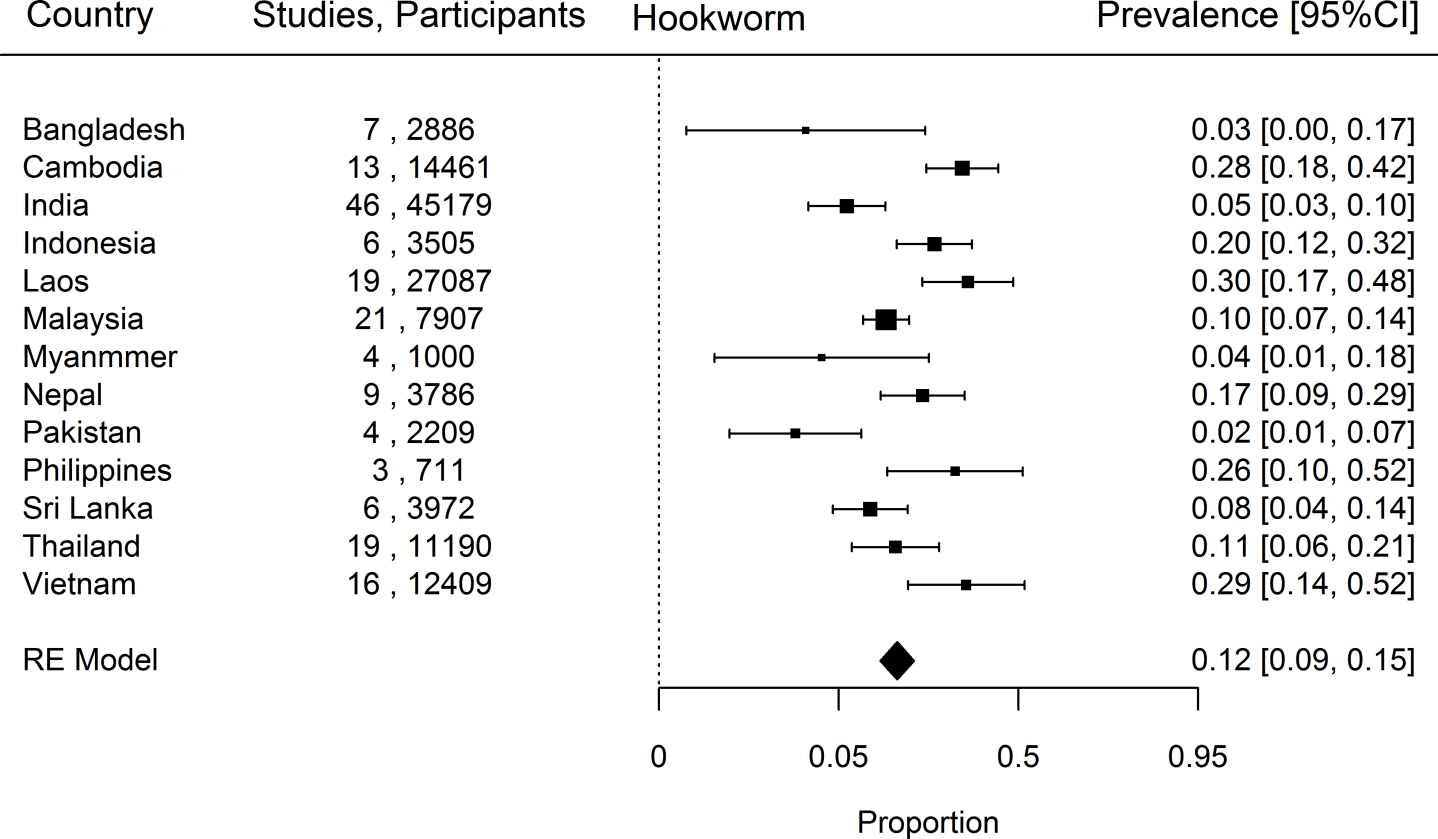
Prevalence of Hookworm in South and South East Asia by Country.

**Fig 3:**
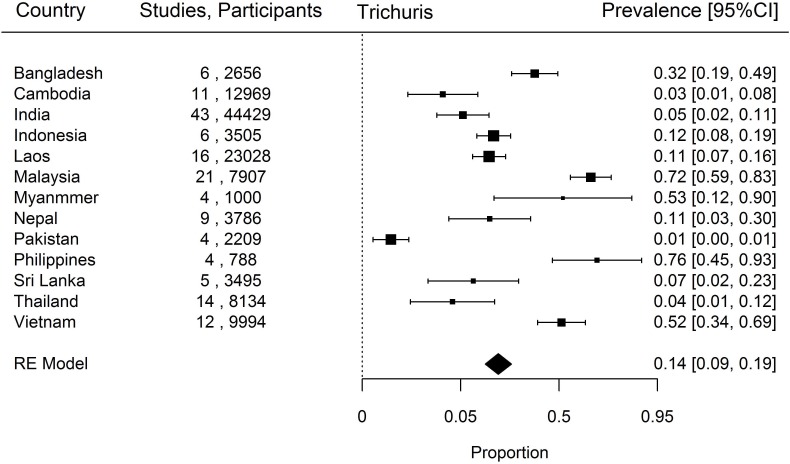
Prevalence of *Trichuris* in South and South East Asia by Country.

**Fig 4:**
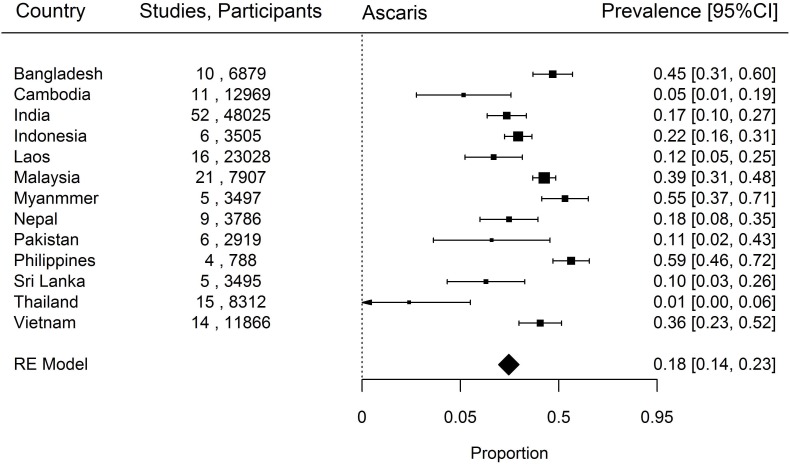
Prevalence of *Ascaris* in South and South East Asia by Country.

When prevalence of STH in the south Asia and south east Asia region was estimated, *Ascaris* was the commonest STH identified with a prevalence of 18% (95% CI, 14–23%) followed by *Trichuris* (14%, 9–19%) and hookworm (12%, 9–15%). The country-specific prevalence of hookworm, *Trichuris* and *Ascaris* have been represented in Figs 2–4. Considerable heterogeneities between and within countries were noticed in the prevalence of STH for all species limiting further statistical analysis (I^2^>90%).

### Hookworm

Among the countries included in our analysis, Laos, which contributed 19 studies and 27,087 participants had the highest proportion of individuals infected with hookworm (Fig 1) (30%, 17–48%), closely followed by Vietnam (16 studies, 12049 participants, 29%, 14–52%) and Cambodia (13 studies, 14461 participants, 28%, 18–42%) (Fig 2). The countries with the lowest proportion of individuals infected with hookworm were Pakistan (4 studies, 2209 participants, 2%, 1–7%), Bangladesh (7 studies, 2886 participants, 3%, 1–17%) and Myanmar (4 studies, 1000 participants, 4%, 1–18%). The wide heterogeneity in prevalence of hookworm infection between countries is reflected on the I^2^ statistic value of 99.6%.

### Trichuris

The highest proportion of individuals infected with *Trichuris* was found in Philippines (4 studies, 788 participants, 76%, 45–93%) and Malaysia (21 studies, 7907 participants, 72%, 59–83) (Fig 3). These prevalence rates were much higher than those found in other countries. The lowest prevalence of *Trichuris* was found in Pakistan (4 studies, 2209 participants, 1%, 0–1%). The between-country I^2^ statistic value for *Trichuris* prevalence was 99.7%.

### Ascaris

Based on the results of our analysis, *Ascaris* is quite widespread throughout south Asia and south east Asia (Fig 4). Myanmar (5 studies, 3497 participants, 55%, 35–71%) and the Philippines (4 studies, 788 participants, 59% 46–72%) were found to have the highest proportion of individuals with ascariasis. Thailand had a notably lower proportion of *Ascaris* compared to the other countries in south east Asia (15 studies, 8312 participants, 1%, 0–6%). The between-country I^2^ statistic value for *Ascaris* prevalence was 99.7%.

### Prevalence of STH infection among different community settings

An important objective of our study was to observe the effect of community setting on the prevalence of STH infection. Studies from rural communities ranged from 84 (with 76,580 participants) for *Trichuris* to 102 (88,790 participants) for hookworm while studies from tribal communities ranged from 28 for *Trichuris* (17,287 participants) to 31 (17257 participants) for *Ascaris*. Studies from urban areas ranged from 53 for *Ascaris* (38, 001 participants) to 42 (30,494 participants) for hookworm.

**Fig 5:**
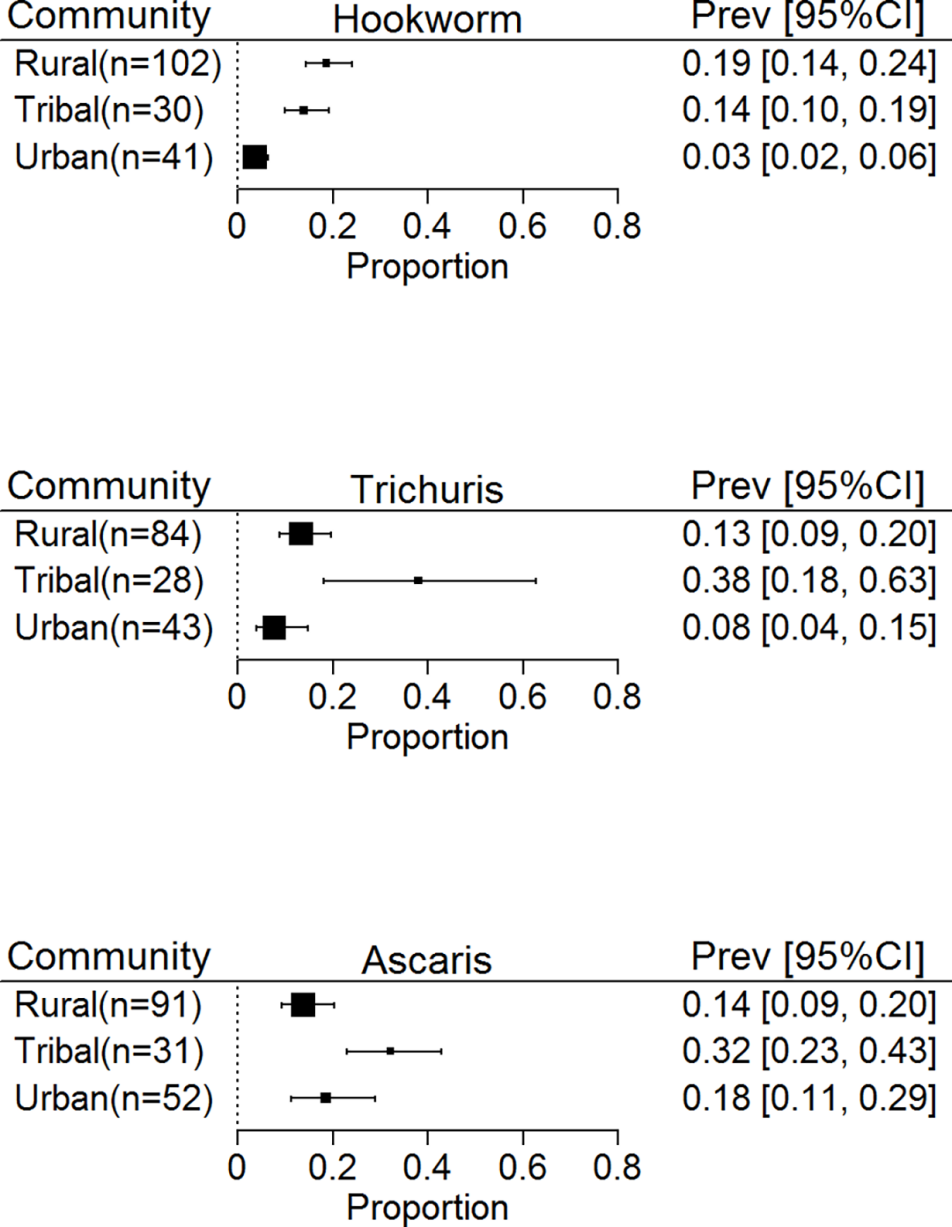
Prevalence of soil transmitted Helminths in South and South East Asia by community type in the general population.

When the effect of community type was examined for the different STH species (Fig 5), prevalence rates of hookworm seen in rural (19%, 14–24%) and tribal communities (14%, 10–19%) were comparable. Tribal communities, however, showed a higher prevalence of *Trichuris* (38%, 18–63%) and *Ascaris* (32%, 23–43%) than rural communities (13%, 9–20% and 14%, 9–20% respectively). Urban communities had much lower rates of hookworm (3%, 2–6%).

### Prevalence of STH infection among school aged children

**Fig 6:**
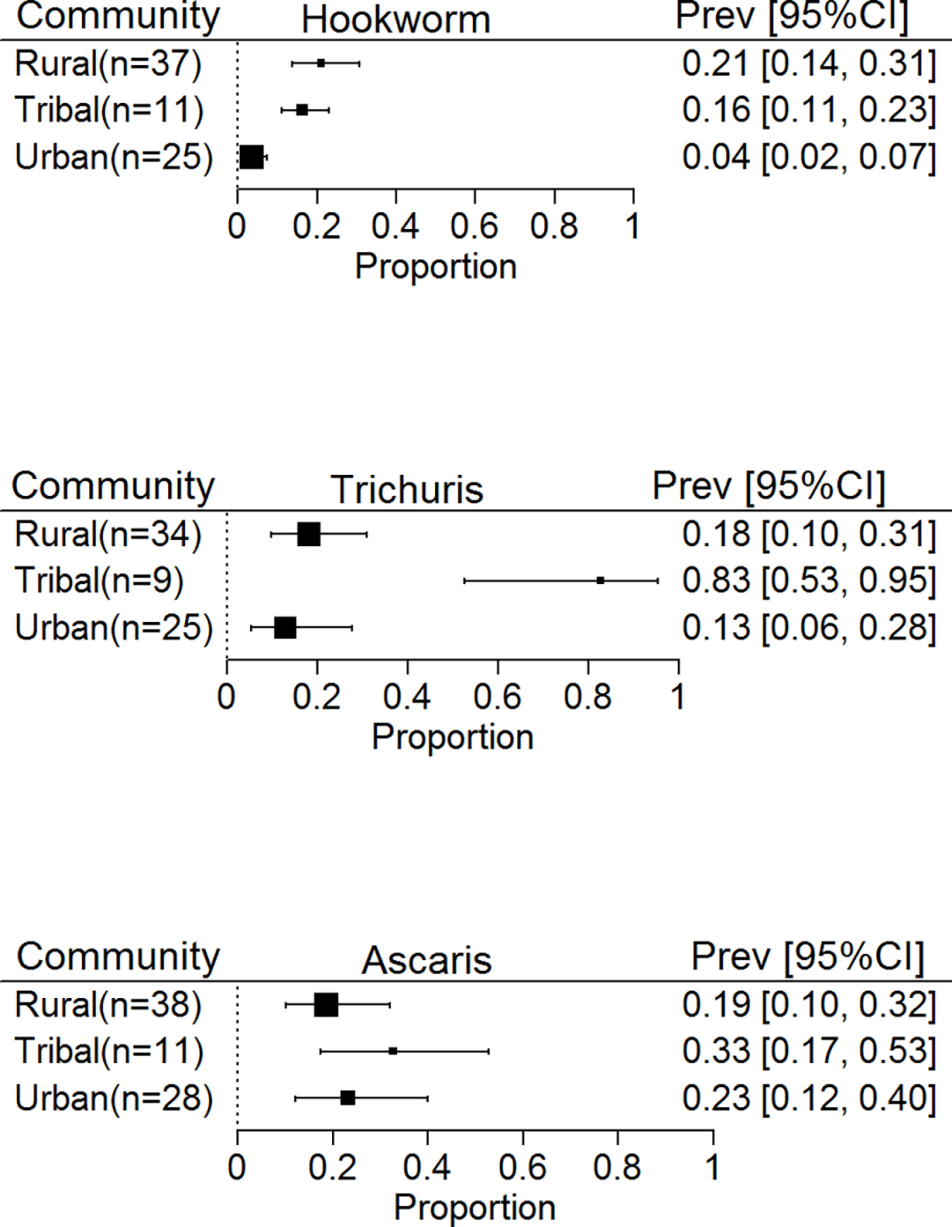
Prevalence of soil transmitted Helminths in South and South East Asia by Community type in School Aged Children.

We also interrogated the studies in our database to determine the prevalence of STH infection among SAC. The prevalence of *Ascaris* (25% 16–31%) and *Trichuris* (22%, 14–34%) were slightly higher among the SAC than among the general population. On the other hand, the proportion of SAC infected with hookworm (10%, 7–16%) was comparable to what was observed in the general population. When analysed by the community setting, the relative proportion of SAC infected with hookworm in the different community settings mirrored the proportions for all individuals living in those settings with higher prevalence seen in rural (21%, 14–31%) and tribal (16%, 11–23%) communities than urban (4%, 2–7%). For *Trichuris*, SAC showed a comparable prevalence in urban (13%, 6–28%) and rural communities (18%, 10–31%) and much higher prevalence in tribal communities (83%, 53–95%) while for *Ascaris*, prevalence was similar in all 3 communities (Fig 6).

## Discussion

Given that STH infections are highly prevalent across south Asia and south east Asia [2], and that several large initiatives have been launched to interrupt transmission [20], we conducted a systematic review of STH infection to identify the communities in south Asia and south east Asia that will benefit the most from intervention programs. Although *Ascaris* was the most prevalent STH in this region, our country specific analysis suggests considerable geographical variation in the distribution of STH. A meta-analysis from South America showed a similar overall high burden of ascariasis [21]. However, in a meta-analysis study from sub-Saharan Africa, hookworm was found to be the commonest STH infection with ascariasis a distant second [22]. Geographical variation in STH infection reflects potential differences in the prevailing climatic and environmental conditions that facilitate transmission of one helminth species over the others. Earlier studies have shown that the abundance and species mix of STH infection can be affected by environmental parameters including surface temperature, rainfall, altitude and soil-type [10, 23–25].

*Ascaris* and *Trichuris* have most often been found in urban and peri-urban communities whereas hookworm are found more often in rural communities [26]. In our analysis, *Ascaris* was found to be the most predominant species in the urban areas as expected, however, in rural areas, *Ascaris*, *Trichuris* and hookworm were present in near equal proportions both in the general population and SAC, suggestive of a greater heterogeneity in the distribution of STH species. Tribal communities, had the highest prevalence of *Ascaris* and *Trichuris* while prevalence of hookworm was comparable with that of the rural population. This high worm burden in the tribal communities reflects their poor and marginalized status, which makes them more vulnerable to STH infections [27].

The current WHO recommendation for STH control is to treat pre-SAC and SAC as they are considered to be at the highest risk of infection [9]. In our analysis, as expected, the prevalence of ascariasis and trichuriasis was higher among the SAC than among the general population, reflecting the age-intensity profile of infection, whereas for hookworm infections, the prevalence among SAC was comparable to that of the general population, suggesting the presence of adult reservoirs of infection [28]. In the absence of improvements in water quality, sanitation and hygiene (WASH) practices, infected and untreated adults are more likely to sustain the community transmission of STH and thereby reduce the overall impact of targeted school based treatment. A modelling-based study on STH transmission has demonstrated that impact of MDA is highly sensitive to the continuous presence of infective larvae in the environment [29].

The non-availability of high quality epidemiological data on STH due to the lack of nationally-representative sample surveys, as well as differences in study design and sampling strategy, have greatly restricted our ability to understand the true prevalence [30]. The wide heterogeneity between studies has restricted our ability to perform more in-depth comparisons. While countries like India had sufficiently large sample sizes, estimates for countries like Afghanistan, Philippines and Myanmar may not be generalizable to the whole country. Moreover, it is quite possible that many of the studies included here were done in high-risk communities, thereby inflating the country-specific prevalence estimates. The unexpectedly high *Trichuris* burden observed in the tribal communities is probably an artifact as a large number of tribal studies were from the Orang Asli community in Malaysia with a very high prevalence of *Trichuris* (9 of the 28 tribal studies were from this community). A proper assessment of the risk of STH will require robust country-specific data, preferably from nationally-representative epidemiological surveys in various communities [30].

In this analysis, we included studies published between 1990 and 2015 –a period spanning 25 years (S1 Fig). Due to changes in social and environmental conditions over time, the prevalence estimates from earlier studies may not be reflective of the current infection status. Additionally, we included only the studies published in PubMed Central or those available through the GAHI database. As a result, we inevitably have missed some studies, especially those published in non-indexed journals, although it is unlikely, given the paucity of data, that their inclusion would have significantly altered the overall findings.

The estimates of STH burden can be affected by the choice of the diagnostic test [31] as well as the number of samples tested per individual [32]. We were not able to adjust for either in the analysis. Moreover, as most studies reported prevalence data, we were only able to estimate the prevalence and not the intensity of infection in these communities. Given the non-linear relationship between the worm aggregation and prevalence [33], this may not be a good indicator of the true disease burden. Also, the impact of treatment is better measured through a reduction in intensity rather than the prevalence of infection [34, 35]. We urge that most future studies publish information on both prevalence and intensity of infection for a better assessment of the community disease burden.

Despite these limitations, this systematic review provides valuable insight into the epidemiology of STH infections in south Asia and south east Asia. The geographical diversity in species distribution demonstrates the need for a flexible approach to effectively control STH infection in this region. While communities with high ascariasis could continue with the school-based deworming approach, those with predominant hookworm infection may have to adopt a population-based deworming approach. Additionally, *Trichuris* endemic communities would require dual therapy [36]. Given the region’s high prevalence of *Ascaris* and *Trichuris*–both of which are transmitted directly by the fecal-oral route [37]–long-term control of STH in these communities will require a multi-faceted approach that also involves improvements in water supply and sanitation.

## Supporting information

S1 Table Table of references used for data extraction on prevalence of soil transmitted Helminths in South Asia (n = 174). (XLSX)

S1 Text PRISMA checklist. (DOC)

S1 Fig Time trend of soil transmitted Helminths Prevalence in South and South East Asia by community type (1990–2015). (TIF)
