# Supplementary figures and images for: Geographical distribution of soil transmitted helminths and the effects of community type in South Asia and South East Asia – A systematic review
Source: PLoS Negl Trop Dis. 2018 Jan 18;12(1):e0006153. doi: 10.1371/journal.pntd.0006153 (PMC5773013; doi:10.1371/journal.pntd.0006153)

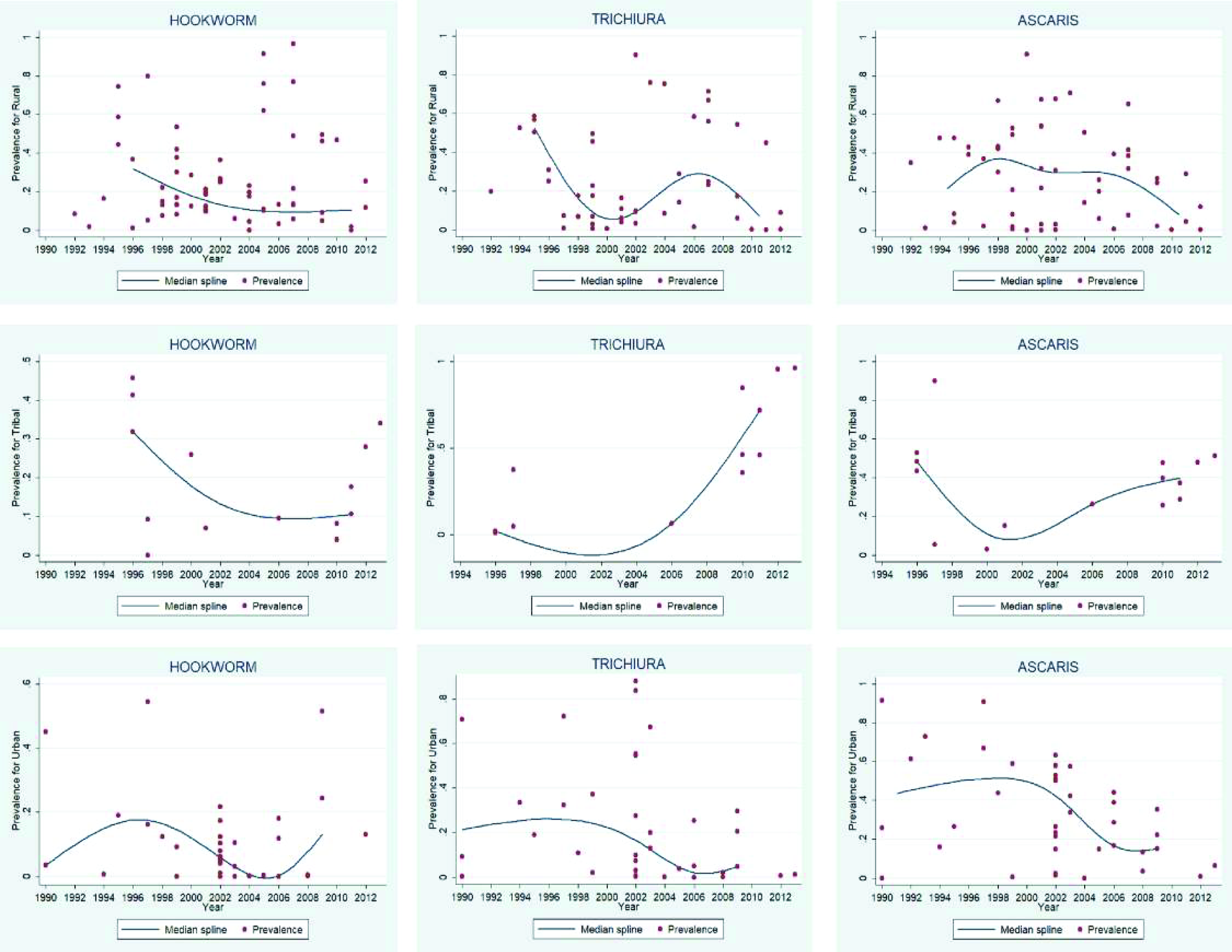

Supplement: S1 Fig — (TIF) [file pntd.0006153.s003.tif]
